# Resolving the 21st century temperature trends of the upper troposphere–lower stratosphere with satellite observations

**DOI:** 10.1038/s41598-023-28222-x

**Published:** 2023-01-24

**Authors:** Florian Ladstädter, Andrea K. Steiner, Hans Gleisner

**Affiliations:** 1 Wegener Center for Climate and Global Change, University of Graz, Graz, Austria; 2 Danish Meteorological Institute, Lyngbyvej 100, Copenhagen, Denmark

**Keywords:** Atmospheric science, Climate change

## Abstract

Historically, observational information about atmospheric temperature has been limited due to a lack of suitable measurements. Recent advances in satellite observations provide new insight into the fine structure of the free atmosphere, with the upper troposphere and lower stratosphere comprising essential components of the climate system. This is a prerequisite for understanding the complex processes of this part of the atmosphere, which is also known to have a large impact on surface climate. With unprecedented resolution, latest climate observations reveal a dramatic warming of the atmosphere. The tropical upper troposphere has already warmed about 1 K during the first two decades of the 21st century. The tropospheric warming extends into the lower stratosphere in the tropics and southern hemisphere mid-latitudes, forming a prominent hemispheric asymmetry in the temperature trend structure. Together with seasonal trend patterns in the stratosphere, this indicates a possible change in stratospheric circulation.

## Introduction

The accumulation of greenhouse gases (GHG) in the atmosphere not only heats Earth’s surface, but also strongly affects the atmosphere itself. Surface temperature trends can be determined with relatively high accuracy due to a large number of long-standing observational records and successful homogenization efforts^1^, and with overall consistency between products, although uncertainties remain^2^. The detailed structure of upper-air temperature trends is more demanding to determine. For decades, observations of upper-air temperature trends have either been of poor vertical resolution, or of limited horizontal coverage.

In the first case, the layer-averaged measurements from the Microwave Sounding Unit (MSU) and its follow-on instruments can hardly resolve the important details around the tropopause, the upper troposphere–lower stratosphere (UTLS) region.

In the second case, the long time series of radiosonde measurements is able to provide useful information also about the UTLS. However, its coverage of Earth’s surface is mostly limited to regions over land, and the carrying balloons often burst before reaching higher altitudes.

In both cases, changes of instrumentation have introduced problems in retrieving robust trend estimates from these observational sources, an issue which has been extensively documented^3–5^.

As a result of discrepancies between temperature trends from various observational systems and data versions, and also between modeled and observed trends, there has been a considerable scientific and also political dispute about the extent of temperature change in the atmosphere^6–9^. For example, tropical tropospheric trends from MSU are smaller than expected from basic physical principles^10^. Santer et al. conclude that “additional independently monitored constraints” are needed to reduce the current large uncertainties of tropical climate change^10^. This situation is unfortunate, since the importance of a detailed knowledge of the UTLS characteristics for understanding the troposphere-stratosphere exchange is undisputed.

The UTLS exhibits strong vertical gradients in atmospheric parameters such as temperature or ozone concentrations. These have been challenging to resolve in both observations and models. Until recently, most weather and climate models have had only a coarsely resolved stratosphere, which was considered sufficient given the low atmospheric mass of the stratosphere. The modeling community has been aware of the importance of the stratosphere–troposphere coupling and its relevance for global circulation and variability, and efforts are undergoing to improve stratospheric representation in models^11^. Recent research points to the strong impact of the stratosphere on the troposphere and even surface weather and climate^12^. Being able to resolve the stratosphere–troposphere coupling is key to a better understanding of those impacts.

The tropical tropopause layer (TTL) as the border between the two regimes is of particular interest. This is the region where the amount of water vapor entering the stratosphere is controlled, mainly by the temperature of the cold point tropopause and by the strength of the tropical upwelling^13^. The stratospheric water concentration plays a main role in determining climate sensitivity^14^ and feeds back on the TTL^15^. The tropical upwelling manifests the upward branch of the stratospheric overturning circulation, also known as Brewer–Dobson circulation (BDC). The BDC is the main meridional air transport mechanism in the stratosphere, and crucial to the understanding of troposphere-stratosphere exchange^12,13,16^. Tropical upwelling has a strong impact on temperatures in the lower stratosphere (LS) region including the TTL through adiabatic cooling and vertical ozone transport^13^. The amount of ozone transported into the stratosphere largely determines the temperature of the tropical LS. Stratospheric temperature and ozone are strongly correlated across a range of time scales due to radiative heating^17^.

In the LS, the partial recovery of ozone due to the Montreal protocol led to weaker cooling trends there since around 1998^6,18^. However, there is evidence that ozone has continued to decline in the LS during the last two decades^19,20^. Interacting with ozone and temperature, the Quasi-Biennial Oscillation (QBO) is the leading mode of inter-annual variability in the stratosphere. The presence of such variability makes robust trend determination an involved task. The vertical structure of temperature trends is therefore often regarded a key uncertainty.

In the troposphere, a shift of the boundary between the tropics and the extratropics, also referred to as the widening of the Hadley cell, is a widely debated change pattern in the climate system^21^. Polewards of this boundary, the upper-tropospheric jet streams are dominating weather patterns. Stratospheric ozone changes and related temperature variations, especially the changing temperature contrast between equator and pole in the UTLS, are postulated to be major drivers of the tropical expansion^22^, with far-reaching consequences for mid-latitude regional weather.

In this work, we present observational evidence of exceptional temperature trends and structural changes in the UTLS over the recent two decades. We focus on providing observational temperature trend information at enhanced spatial resolution, considering monthly and seasonal variability.

The observational groundwork to achieve this is laid out by recent advances in satellite measurement technique. Since the beginning of the 21st century, Global Navigation Satellite System (GNSS) radio occultation (RO) measurements are available, with the potential to drastically increase knowledge about the thermal structure of the UTLS. The limb-sounding geometry of RO yields a particularly high vertical resolution of about 100 m around the tropical tropopause, together with full global coverage. For trend analysis, many properties of RO are highly beneficial, among those the long-term stability, and the ability to merge time series of different RO missions without homogenization, due to the measurements being based on highly-precise atomic clocks^23,24^. Uniquely for GNSS RO, information on altitude and pressure are derived independently^25^. This allows trends in geophysical parameters to be determined without being intertwined with trends in the height of constant pressure surfaces.

The unique ability of GNSS RO to resolve steep vertical gradients enables us to explore fine details of the trend structure around the tropopause. This has also been acknowledged by the latest Intergovernmental Panel on Climate Change (IPCC) Assessment Report (IPCC AR6), which for the first time includes results from RO in the section on upper-air climate^26^. Previous studies on temperature trends from GNSS RO have focused on intercomparisons between different datasets, and have analyzed shorter time periods^5,27–29^. In this study we improve on previous work by using 20 years of observational data, providing trend values on the altitude coordinate, independent of trends in atmospheric pressure.

## Methods

We use GNSS RO temperature profiles processed by the Radio Occultation Meteorology Satellite Application Facility (ROM SAF)^30^. Due to the low structural uncertainty of RO data in the UTLS, the choice of RO data processing has only a negligible effect on the trend results^31^.

Here we use the so-called dry temperature, which equals physical temperature in the UTLS. RO dry temperature is a retrieved variable closer to the measurement than RO physical temperature. The latter is the result of combining measurement with background data, relevant in the lower troposphere where humidity is not negligible^23,24,31–33^.

The individual profiles are aggregated to monthly means at $5^{\circ }$ latitudinal resolution, as well as to $2.5^{\circ }$ × $2.5^{\circ }$ gridded records for investigating regional trends. In the latter case, each grid box contains all profiles within 300 km of the center point, such that each grid box represents the same area on Earth’s surface. To minimize errors due to time-varying sampling of the atmosphere^4,34^, the sampling error is estimated and subtracted using a global background field (ERA5.1^35^). From the resulting monthly mean fields, temperature anomalies are created by subtracting the mean seasonal cycle.

The complex variability structure of the UTLS, combined with a rather short time period of about 20 years, requires careful trend determination techniques. Two characteristics of the regression residual require special attention: First, values of the residual time series are not statistically independent, but exhibit a temporal autocorrelation, which, in a naive least squares linear regression approach, lead to an inflated estimate of the significance of trend results^36^. In our case, the monthly temperature anomalies time series exhibit a predominant autocorrelation of lag one (one month). For that, an autoregressive model of order 1 (AR[1]) can be used to properly account for the autocorrelation. Second, and related to the first, quasi-periodic features such as the QBO remaining in the residual also violate the required properties of being uncorrelated and normally distributed^37^.

The different states of the QBO in the stratosphere, and the El Niño–Southern Oscillation (ENSO) in the troposphere and tropopause region, both have a decisive impact on atmospheric variability. Because of that, they might affect long-term trend estimates. Using proxies for these phenomena in a multiple regression formulation, the influence on the resulting linear trend estimate can be minimized. For QBO and ENSO, wind indices and sea surface temperature are commonly used as proxies.

We employ an AR(1) model in a multiple regression formulation. As regressors, the first three components of a principal components analysis over the monthly mean zonal wind components for all 15 available pressure levels of the Singapore wind data are used to account for the QBO. In addition, an ENSO index from monthly sea surface temperatures with a time lag of three months is used.

## Results

**Figure 1:**
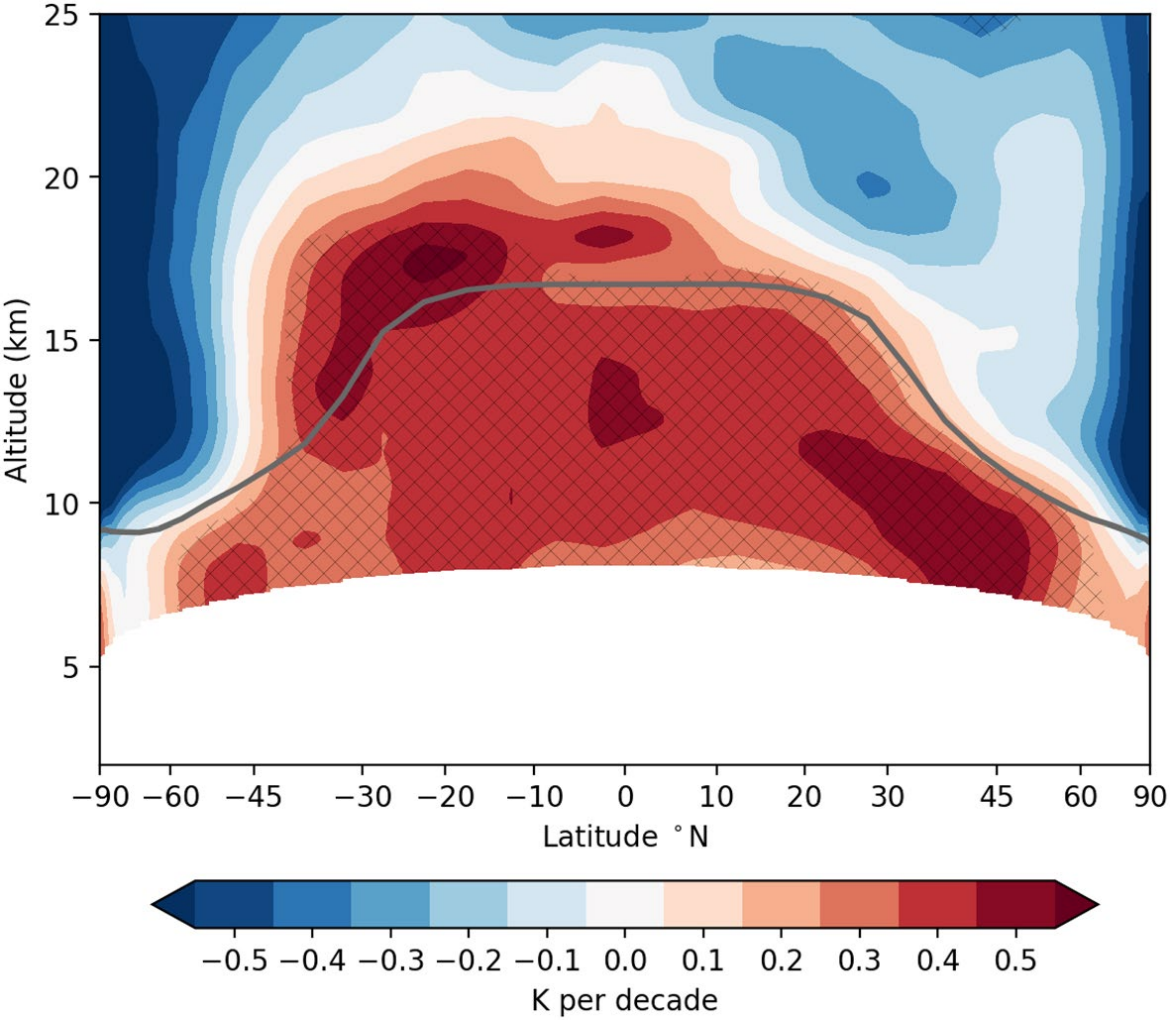
Observed global atmospheric temperature trends in the UTLS from January 2002 to December 2021, with a latitudinal resolution of 5$^{\circ }$. The gray line indicates the height of the tropopause. Areas with trends significant at the 95% level are marked with black crosses.

Within the last two decades alone, upper-tropospheric temperatures have increased by up to 1 K in the tropics and in northern mid-latitudes (Fig. 1). Throughout the upper troposphere, these trends are significant at the 95% level. These concerning numbers support the hypothesis that corresponding MSU trend estimates are likely too small^5,10,38^. For the tropical upper troposphere as an example, MSU trend estimates are smaller by approximately a factor of two compared to GNSS RO (see Supplementary Fig. S1). The MSU measurements contain averaged information for a broad height layer, including regions with smaller temperature changes^5^. Part of the underestimation by MSU can also be explained by drifting orbits^38^.

In the tropics and the SH, the warming extends through the tropopause into the LS, reaching up to about 20 km in SH mid-latitudes. This results in a prominent hemispheric asymmetry of LS temperature trends. A similar structure has already been described before, but for a shorter time period^29^. In the northern hemisphere (NH), the atmosphere is cooling above the tropopause, particularly in sub-tropical regions around 20 km. In the tropics, the transition between warming and cooling lies around 22 km, with increasingly negative trends above.

Previous work on LS temperature trends has found near-zero post-millennium trends in the tropics, in contrast to the decades before 2000 with a distinct cooling signal^5,6,18^. This is attributed to the radiative effect of the recovery of ozone. The observational source for those trend estimates is often MSU and related instruments. For the LS, the MSU channel 4 is used, with layer-averaged information on temperature-equivalents for roughly 13–22 km, with contributions from below and above that. Comparing to Fig. 1 hints towards an incomplete representation of the true LS trends from MSU instruments, averaging over the extended positive TTL and negative stratospheric trend regions^39^. This can also be seen more quantitatively when focusing on mean tropical temperatures (Supplementary Fig. S1). RO is clearly able to present a view on the trend signal with unprecedented details.

**Figure 2:**
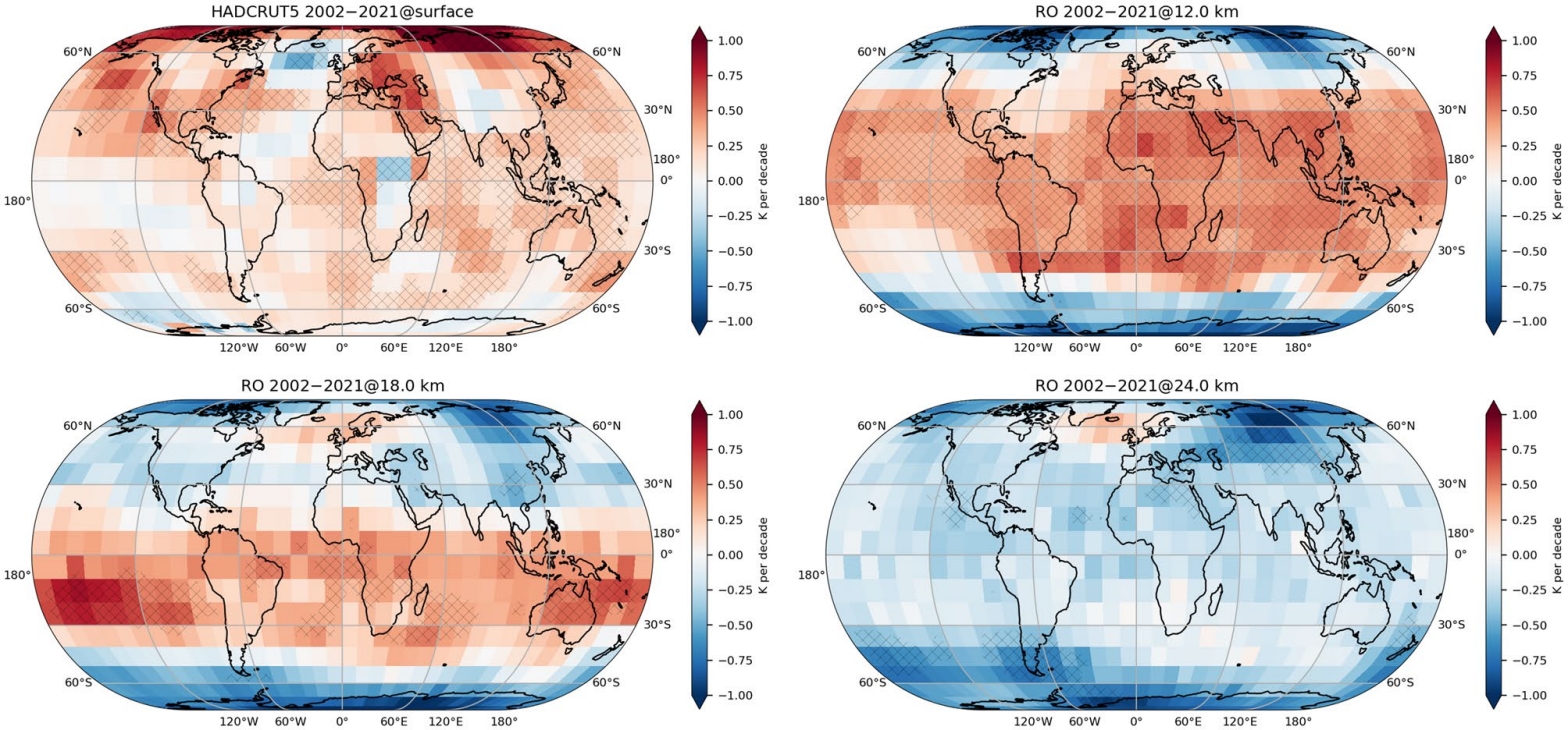
Observed regional temperature trends from the surface to the stratosphere from January 2002 to December 2021, in 10$^\circ$x 10$^\circ$ horizontal resolution. (top left) HadCRUT surface data; GNSS RO data for (top right) 12 km, located in the troposphere in the tropics and stratosphere at high latitudes; (bottom left) stratosphere at 18 km, and (bottom right) stratosphere at 24 km. Areas with trends significant at the 95% level are marked with black crosses. The maps were created using Cartopy v0.20.2 (https://scitools.org.uk/cartopy).

Turning from a detailed vertical perspective to a horizontal, i.e. regionally resolved, view, Fig. 2 cuts through Earth’s UTLS atmosphere at several height levels. In addition, surface trends from one representative global surface temperature product, the HadCRUT5 dataset^1^, over the same time period are shown, to put the upper-air temperature changes in relation to the surface.

At each height level we observe distinct trend patterns. Tropical amplification, the amplified warming of the upper tropical troposphere compared to the surface^40^, is prominently observable, comparing the surface and the 12 km layer. Cooling in the Northern Atlantic region, a well-known feature called the warming hole^41^, continues, and our analysis reveals that this surface cooling is accompanied by a warming in the LS. At 24 km, an altitude where we have predominantly negative temperature trends around the globe, this warming pattern is striking. The region of warming corresponds to a region of positive ozone trends^42^, and is possibly induced by a decrease of the regional tropopause height^43^.

At 18 km, just above the tropical tropopause, and well into the stratosphere outside of the tropics, the most prominent feature is the clear hemispheric asymmetry. The LS is significantly warming in large parts of the tropics and SH, with particularly large trend values in the subtropical SH Pacific. The NH shows no significant temperature signal at most latitudes, except for polar regions with strong cooling, and the “warm blob” over the Northern Atlantic.

The hemispheric asymmetry of stratospheric trends points towards a possible connection to ozone. In the LS, ozone is the major driver for temperature trends, and ozone trends show a strong seasonality^44^.

**Figure 3:**
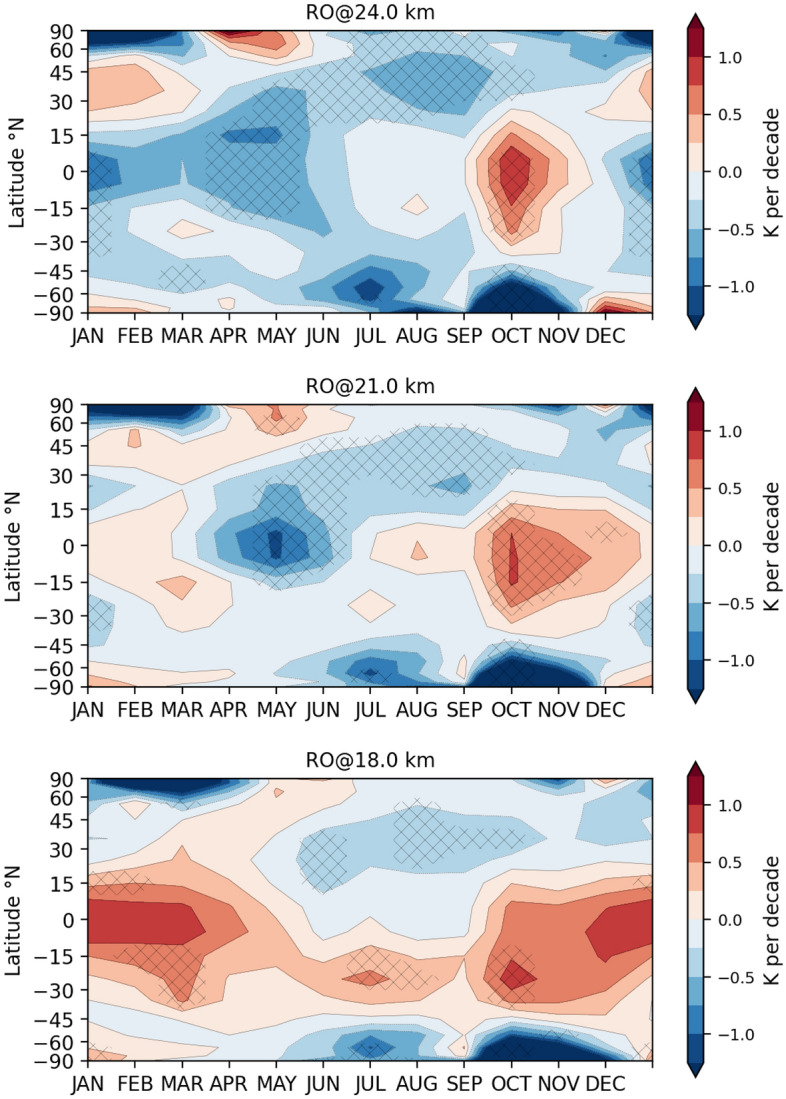
Observational seasonal trends for GNSS RO temperature shown over latitude versus months, for three different stratospheric altitude layers. (top) 24 km, (center) 21 km, (bottom) 18 km. Areas with trends significant at the 95% level are marked with black crosses.

We provide insight into the seasonality of trends in a vertically differentiated picture. The seasonal signature of LS temperature trends in Fig. 3, at 18 km, 21 km, and 24 km, exhibits—somehow surprising—a strong negative temperature trend at SH polar regions in austral spring (October–November). This is in contrast to expected stratospheric warming due to the recovery of ozone^6,18^. The combined pattern of spring cooling of the Antarctic stratosphere and warming of the tropical LS could be a fingerprint of a weakening of the SH branch of the BDC^29,45^. This weakening is not expected from model projections, which show an acceleration of the BDC due to increasing GHG concentrations in the 21st century^46^. However, ozone recovery could have a counteracting effect^16^. The vertical resolution is key here to be able to resolve these change patterns, providing a clear advantage over layer-averaged observations.

In the NH, the opposite effect is observable. Warming in Arctic spring and at the same time cooling in the tropics are a signal for BDC acceleration in the NH.

It is important to note that for the relatively short time period of 20 years, atmospheric variability makes robust trend determination difficult. This variability can be (multi-)decadal such as the Pacific Decadal Oscillation^47^, or short-term such as the strong variability in polar regions. During the investigated time period, two rare SH southern stratospheric warmings (SSW) occurred in 2002 and 2019, imposing the suspicion that the related strong temperature anomalies could tamper with the trend estimates. This is, however, not the case, as the result holds even when removing the SSW time periods from the regression.

## Conclusions

A detailed knowledge about temperature changes in the UTLS, including structural changes and changes in the coupling between troposphere and stratosphere, is fundamental for a better understanding of atmospheric dynamics and its impacts on global and regional climate.

The results indicate substantial changes in the UTLS during the observed time period:

- The troposphere shows a warming of up to 1 K during the first two decades of the 21st century, particularly in the tropics and in northern mid-latitudes.
- The tropopause region reveals a prominent hemispheric asymmetry of changes in the LS temperature patterns.
- Tropospheric warming extents through the tropopause in the tropics and SH mid-latitudes, up to a height of about 20 km.
- Layer-averaged observations miss important details of trend patterns, and likely underestimate temperature trends in the UTLS.
- Regional trend patterns reveal that the surface cooling in the Northern Atlantic region translates into a stratospheric warming at around 24 km over the same region, whereas the rest of the stratosphere is cooling.
- Seasonal variability of temperature shows a combined pattern of Antarctic cooling and tropical warming in the LS in austral spring. This could be a fingerprint of a weakening of the SH branch of the BDC.
- Warming is observed in the LS in Arctic spring, and cooling in the tropical LS at the same time. This could hint towards a BDC acceleration in the NH.

Satellite observations from GNSS RO can substantially contribute to the climate monitoring in the UTLS region of the atmosphere. The high vertical resolution and insight into the fine structure of atmospheric temperature with GNSS RO is defining a new state-of-the-art representation of Earth’s upper-air climate change. We recognize this to be a major advance towards global climate monitoring.

In conclusion, our findings provide observational evidence that the temperature trends in the UTLS have been underestimated in the literature. Our results document a large warming of the tropical upper troposphere, and indications for structural changes in the global circulation patterns. These findings, supported by recent research^45,48–52^, reveal an accelerated change of the global climate in the first decades of the 21st century.

## Supplementary Information

Supplementary Information.

## Data Availability

The GNSS RO profiles can be acquired from the ROM SAF^53^ (doi:10.15770/EUM_SAF_GRM_0002, 10.15770/EUM_SAF_GRM_0003, 10.15770/EUM_SAF_GRM_0004, 10.15770/EUM_SAF_GRM_0005, and data from https://www.romsaf.org/). Surface temperature data was downloaded from the Climatic Research Unit (University of East Anglia, https://crudata.uea.ac.uk/cru/data/temperature/). The Singapore wind data was downloaded from https://www.geo.fu-berlin.de/met/ag/strat/produkte/qbo/, and the ENSO index from https://www.cpc.ncep.noaa.gov/data/indices/.
