# Supplementary material for: Resolving the 21st century temperature trends of the upper troposphere–lower stratosphere with satellite observations
Source: Sci Rep. 2023 Jan 24;13:1306. doi: 10.1038/s41598-023-28222-x (PMC9873623; doi:10.1038/s41598-023-28222-x)
Supplement: Supplementary file 1 — Supplementary Information. [file 41598_2023_28222_MOESM1_ESM.pdf]

Supplementary information

Resolving the 21st century temperature trends of the upper troposphere–lower stratosphere with satellite observations

Authors: Florian Ladstädter(1), Andrea K. Steiner(1), Hans Gleisner(2).
(1) Wegener Center for Climate and Global Change, University of Graz, Graz, Austria
(2) Danish Meteorological Institute, Lyngbyvej 100, Copenhagen, Denmark

Supplementary figures

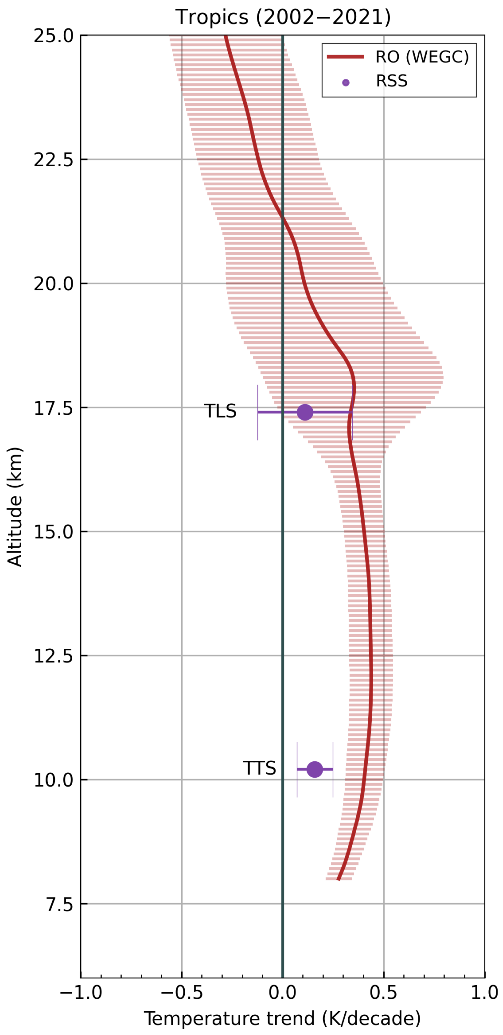

Supplementary Figure S1. Observed atmospheric temperature trends in the UTLS for GNSS RO and (A)MSU (RSSv4.0) from January 2002 to December 2021, for the Tropics (20°S to 20°N). For (A)MSU, the lower stratospheric channel 4/9 (TLS), and the upper tropospheric channel 3/7 (TTS) are shown. The (A)MSU data were downloaded from https://www.remss.com.
